# Preparation and Property of Bio-Polyimide/Halloysite Nanocomposite Based on 2,5-Furandicarboxylic Acid

**DOI:** 10.3390/polym13234057

**Published:** 2021-11-23

**Authors:** Yingxia Chen, Shuya Fan, Xibin Yi, Bing Li, Shiwei Chen, Shuyu Liu, Tao Hu, Si Chen

**Affiliations:** 1 Shandong Provincial Key Laboratory of Processing and Testing Technology of Glass and Functional Ceramics, School of Material Science and Engineering, Qilu University of Technology (Shandong Academy of Sciences), Jinan 250353, China; 13884894032@163.com (Y.C.); 15550913065@163.com (S.F.); shuyu031251@163.com (S.L.); xixixiaohu1234@163.com (T.H.); chensi11233@163.com (S.C.); 2 Shandong Key Laboratory for Special Silicon-containing Material, Advanced Materials Institute, Qilu University of Technology (Shandong Academy of Sciences), Jinan 250014, China; yixb@sdas.org (X.Y.); mindless000cn@163.com (B.L.)

**Keywords:** bio-based polyimide, halloysite nanotubes, thermal property, mechanical properties

## Abstract

Bio-based polyimide (PI)/halloysite nanotube (HNT) nanocomposites based on 2,5-furandicarboxylic acid were prepared by in situ polymerization. The pristine HNTs were modified by tetraethoxysilane (TEOS) and 4,4′-oxybisbenzenamine (ODA). The bio-based PI/HNT nanocomposite film exhibited lower moisture absorption than pure bio-based polyimide, showing that the water resistance of the bio-based polyimide film was improved. The thermal stability and glass transition temperature (Tg) of PI/HNTs nanocomposites were improved with the addition of modified HNTs. Both the tensile strength and Young’s modulus of bio-based PI/HNTs nanocomposite films were enhanced. A 37.7% increase in tensile strength and a 75.1% increase in Young’s modulus of bio-based PI/HNTs nanocomposite films, with 1 wt% of the modified HNTs, were achieved. The result confirmed that 2,5-furandicarboxylic acid could replace the oil-based material effectively, thus reducing pollution and protecting the environment. Finally, a preparation mechanism to prepare bio-based PI/HNTs nanocomposite is proposed.

## 1. Introduction

Polyimide (PI) is a particular kind of polymer, whose molecular chain contains an imide ring. The molecular chain of PI has many aromatic rings and heterocycles. PI shows excellent thermal stability, flame retardant, high insulation, a low dielectric constant, and high mechanical properties [1,2,3,4,5,6]. Notably, PI has the best thermal stability among the polymers and exhibits excellent comprehensive properties in each application field. Film was one of the earliest PI products. It is widely used in aerospace, microelectronics, atomic energy, electrical insulation, liquid crystal display, membrane separation technology, and other fields [7,8,9,10,11]. PI film is known as “gold film” and is the key to influencing the technical development of industries in many countries.

Recently, polyimide film has become more widely available in high-tech fields such as aeronautics and astronautics. These fields need high-temperature resistance of the polyimide film [12,13,14]. With the continuous development of modern industry and the expansion of applicable fields, it is urgent to prepare polyimide films with high-temperature resistance, strength, and modulus [15,16,17]. In order to improve the properties of polyimide films, much research on polyimide composite films has been conducted. The widely used solution was to add inorganic particles to polyimide films [18,19]. Fang prepared a novel PI composite film based on lithium bis(trifluoromethanesulfonyl)imide (LiTFSI). The PI/LiTFSI composite film, containing 15 wt% LiTFSI, displayed comprehensive property [20]. Zhao synthesized ultralong titanate nanotubes, which were used to prepare PI/titanate nanotube composite films. They found that PI/titanate nanotube composites exhibited improved electrical and mechanical properties compared to that of pure PI [21]. Shin prepared two series of transparent polyimide (PI) hybrid films with organically modified clay by solution intercalation polymerization and thermal imidization. They found that hybrids exhibited higher glass transition temperatures (Tg) and mechanical properties, owing to the addition of the organically modified clay [22].

HNTs are naturally layered silicate nanotubes, which are abundantly available. The structure is similar to that of a carbon nanotube [23,24]. Carbon nanotubes are excellent additives in the composites; however, they are too expensive. In contrast, the price of HNTs is much cheaper than that of carbon nanotubes. Thus, HNTs are a good substitute for carbon nanotubes. The specific surface area of HNTs is 50 m^2^/g, the pipe diameter is 8–17 nm, and the pore volume is 0.2–0.4 cm^3^/g. HNTs are novel 1D natural nanomaterials with a unique combination of natural availability, large aspect ratio, rich functionality, tubular nanostructure, high mechanical strength, and good biocompatibility. These characteristics generate exceptional mechanical, thermal, and biological properties for HNT–polymer nanocomposites at a low cost [25]. Therefore, HNTs could be used as good additives in high-performance polymer nanocomposites and multifunctional nanocomposites. In the last decade, HNTs have been widely used as an additive to modify polymers. HNTs can improve the mechanical properties, thermal stability, and flame retardancy of polymers [26,27,28,29,30]. Lisuzzo prepared Mater-Bi/halloysite nanocomposite materials that could be employed as a bioplastic alternative to petroleum-derived products [31]. They found that the HNTs could effectively enhance thermal and tensile performance. Huang prepared halloysite/agar-based nanocomposites and found that the tensile strength and elongation at break of nanocomposites increased remarkably with the increase in chitosan and/or halloysite content [32]. Yang prepared PEO/halloysite nanocomposites. Their result showed that the tensile strength of the nanocomposites was improved effectively compared to that of pure PEO [33]. In general, there are two crucial factors influencing the property of polymer/HNTs nanocomposites: a good dispersion of the HNTs in the polymer matrix and a desirable interfacial affinity between the HNTs and the polymer. Because of the hydrophilic surface and the negatively charged external surface, it remains difficult to achieve a good dispersion of HNTs in the polymer matrix [24,25]. Scientists grafted coupling agents on the surface of HNTs to enhance the dispersion of HNTs in the polymer matrix and interfacial interactions. In addition, Zhang used TEOS as a silica precursor and silane as a surface modifier to modify clay. They found that this silica shell on the surface of the clay had many silanol groups, which greatly promoted silylation of the clay [34].

Today, resource shortage and environmental pollution are becoming more and more serious [35,36,37,38,39]. Meanwhile, with increasing awareness of environmental protection, the industries that consume considerable non-renewable resources or seriously destroy the environment are facing severe challenges [40,41,42]. Research on renewable resources to replace non-renewable resources, such as coal, oil and natural gas, has become a major focal point. Nowadays, governments around the world are making efforts to study and use new energy. Bio-based material refers to products existing in the biological world. With global joint efforts, they have become a renewable resource with the highest global output [43,44,45,46]. Among them, 2,5-furandicarboxylic acid is the only biological monomer with a five-membered ring, which makes it the strongest and most structurally stable among the bio-based monomers [47,48,49]. Bio-based polymers based on 2,5-furandicarboxylic acid have attracted more and more attention from scientists.

Therefore, it is urgent to develop a bio-based polyimide composite film which can replace petroleum-based monomers, and which has excellent comprehensive properties.

Herein, we propose a new and effective approach to fabricate the bio-based PI/HNTs nanocomposite based on 2,5-furandicarboxylic acid via in situ polymerization. The study aims to prepare bio-based polyimide/halloysiste nanocomposite film for the first time. We expect to use the bio-based monomer to replace oil-based material, which could reduce environmental pollution. In addition, we want to improve the thermal stability and tensile property of bio-based polyimide film.

## 2. Materials and Methods

### 2.1. Materials

HNTs were supplied by SanXing High-New Material Company of Zaozhuang, China. 2,5-furandicarboxylic acid, 3,3,4,4-benzophenone tetracarboxylic dianhydride (BTDA) and ODA were supplied by Alfa Aesar, Shanghai, China. Dimethyl sulfoxide (DMSO), Et_3_N, TEOS, ethanol, trimethylamine (Et_3_N), acetic anhydride, and N, N-dimethylacetamide (DMAc) were all reagent grades and provided by Beijing Chemical Reagents Company, Beijing, China. Deionized water was used in all experiments.

### 2.2. Preparation of Modified HNTs

Firstly, the dried HNTs (10.0 g) were dispersed in the mixtures of ethanol (100.0 mL) and ammonia (18.4 mL). The solution was kept stirring for 2 h at room temperature. Then the temperature was adjusted to 60 °C and TEOS (2.0 mL) was added with continuous stirring for 6 h. Subsequently, the slurry was filtered, washed with ethanol several times, and dried at 110 °C for 12 h.

Secondly, the product above (10.0 g) was dispersed in the ethanol (100.0 mL) again and stirred for 2 h. Meanwhile, ODA (1.0 g) was added into the slurry and was kept stirring for another 2 h at room temperature. Then the slurry was filtered, washed with ethanol several times, and dried at 110 °C for 12 h. The obtained power was the modified HNTs and was termed m-HNTs.

### 2.3. Preparation of Bio-Based Diamine

Firstly, 2,5-furandicarboxylic acid reacted with DMSO to prepare 2,5-furandicarbonyl dichloride according to the method reported by Ma et al. [50].

Secondly, DMAc (30 mL), Et_3_N (24 mmol), and ODA (24 mmol) was added in a three-necked flask and stirred for 15 min at room temperature. Under the condition of a N_2_ atmosphere, 2,5-furandicarbonyl dichloride (20 mmol) in DMAc (20 mL) was added dropwise to this solution. After being stirred for another 24 h, the mixture was poured into the water. Then, the precipitate occurred. Subsequently, the precipitate was filtrated, washed with water several times, and dried under a vacuum at 90 °C. The product was named FDCA-OA and the product yield was 70%.

### 2.4. Preparation of Bio-Based PI/HNTs Nanocomposite

m-HNTs (0.05 g) were dispersed in the DMAc (10.0 mL) and stirred for 2 h. A 100 mL three-necked flask equipped with a nitrogen inlet tube and a mechanical stirrer was added with BTDA (1.90 g) and DMAc (20.0 mL). The solution was kept in N_2_ atmosphere for 1 h. A homogeneous solution could be obtained when BTDA was completely dissolved. Bio-based diamine FDCA-OA was added into the flask and stirred for 3 h. Subsequently, the mixtures of the m-HNTs and DMAc were put into the flask and stirred for 4 h. Finally, a viscous and brown solution was obtained and subsequently used to prepare the films.

### 2.5. Preparation of Bio-Based PI/HNTs Nanocomposites Films

The solution above was sonicated for 30 min to remove gas bubbles. Then, the solution was poured into a clean glass plate and dried at 60 °C for 12 h to evaporate the DMAc. Subsequently, the solution was step-cured (at each temperature of 100 °C, 200 °C, 250 °C, and 300 °C for 1 h). After curing, light yellowish films were obtained.

The films including m-HNTs 1 wt%, 3 wt%, and 5 wt% were denoted as PI-HNTs-1%, PI-HNTs-3%, and PI-HNTs-5%, respectively. To make the comparison, pure bio-based PI film was also prepared and named PI. The pristine HNTs were used to prepare the film, which was named PI-PHNTs-1% when the content of the pristine HNTs was 1 wt%.

### 2.6. Characterization

The X-ray diffraction (XRD) spectra of all samples were obtained in a Siemens D-500 diffractometer with the angle 2*θ* from 10° to 70°. The layer distance of HNTs could be obtained using the Bragg equation:

(1) $$d=\frac{n\lambda }{2\sin\theta }$$

where *n* is an integer determined by the order given, *λ* is the wavelength of the CuKα radiation source, and *θ* is the angle. The microstructures of the samples were characterized in an FEI XL 30 scanning electron microscope (SEM) (Hillsboro, OR, USA) at 20 kV acceleration voltage. Fourier-transform infrared (FTIR) spectra were obtained in a Spectrum 1000 Perkin-Elmer spectrometer (Waltham, MA, USA) in the spectral area of 400–4000 cm^−1^. Specimens were prepared by grinding the sample with potassium bromide (KBr). Moisture absorption measurements of polyimide nanocomposite films (10 ± 2 um) were carried out in an environmental chamber at 30 °C for 72 h under 85% relative humidity. Specimens were cut into squares of 5 × 5 cm^2^, annealed in an oven at 80 °C for 24 h, and cooled down to room temperature. The percentage of moisture absorption was calculated by the increase in weight divided by the original specimen weight. Thermogravimetric analysis (TGA) was carried out for the samples under nitrogen purge at a heating rate of 20 °C/min from 25 °C to 800 °C using a TG-DTA apparatus(STA449, Netzsch, Selb, Germany). Differential scanning calorimetry (DSC) was conducted by DSC SDT Q600 (TA, New Castle, DE, USA)with a heating rate of 10 °C/min from 100 °C to 390 °C under N_2_ atmosphere to obtain the glass transition temperatures (Tg) of the samples. The measurement of the mechanical properties of films were tested by Instron testing machine Model 55 67 according to ASTM D638. Average values of five specimens for each sample were reported.

## 3. Results and Discussion

### 3.1. The Preparation Scheme of Bio-Based Polyimide/HNTs Films

**Figure 1:**
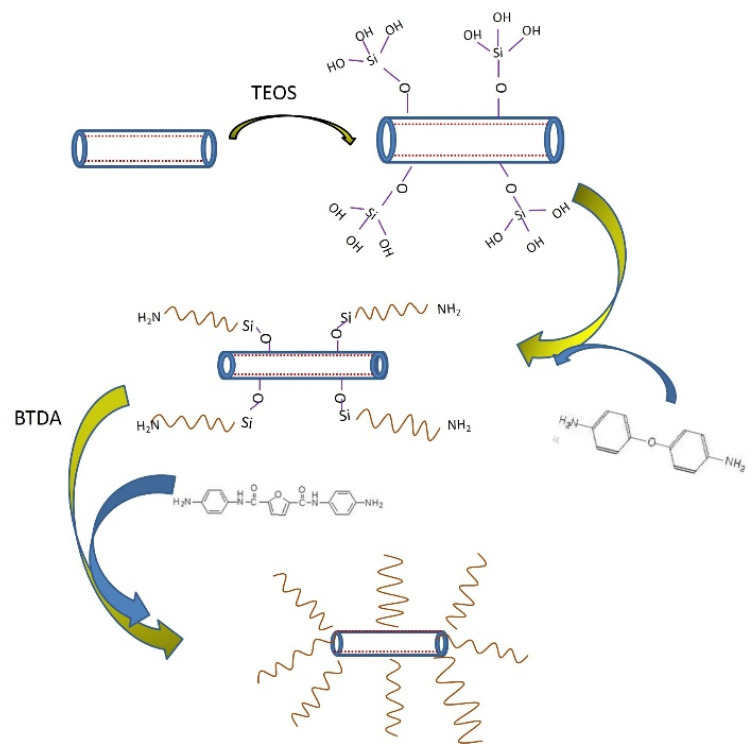
The preparation scheme to prepare bio-based polyimide/HNTs nanocomposite.

The scheme to prepare bio-based polyimide/HNTs nanocomposite is illustrated in Figure 1. Owing to the hydrolysis of TEOS, more OH groups covered the surface of the HNTs. These OH groups could have a chemical or physical interaction with the NH_2_ groups of the ODA. TEOS made the ODA modify the HNTs more efficiently. BTDA could have had a pre-polycondensation reaction with bio-based diamine to obtain linear polyamic acid. When the temperature went up to 150 °C, polyamic acid could have had a cross-linking reaction to obtain ring polyimide. Due to the polar groups, m-HNTs could have had a chemical or physical interaction with the polyimide. Thus, the compatibility and interfacial interaction between m-HNTs and polyimide matrix was improved, leading to strong structural stability and effective stress transfer. Finally, the improved tensile strength and Young’s modulus of bio-based PI/HNTs nanocomposite film could be expected, compared to that of pure bio-based PI.

### 3.2. Characterization of m-HNTs

Halloysite is a hollow cylinder with a basal spacing (d_001_) of 1 nm. Halloysite has an internal surface composed of aluminol (Al-OH) groups and an external surface composed of siloxane (Si–O–Si) groups. A monolayer of water molecules is weakly held in the interlayer of halloysite. When the halloysite is dehydrated, the basal spacing of the interlayer can be reduced to 0.73 nm [51].

**Figure 2:**
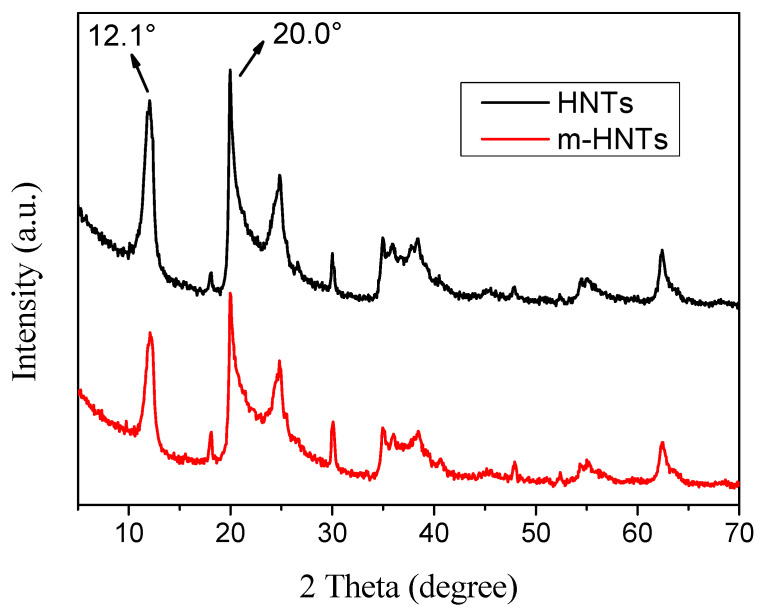
XRD patterns of HNTs and m-HNTs.

Figure 2 shows the XRD patterns of HNTs and m-HNTs. The typical diffraction peaks (d_001_) at 2*θ* = 12.1° occurred in the spectrum of both HNTs and m-HNTs. According to the Bragg equation, the layer distance of two samples was 0.73 nm. The typical diffraction peak on the spectrum was still at the same position, indicating that the TEOS and ODA did not insert into the interlayer space of HNTs. It is noted that the peak intensity (especially 2*θ* = 12.1° and 20.0°) of m-HNTs decreased compared to HNTs. This could be attributed to the TEOS and ODA covered on the HNTs. The result showed that TEOS and ODA were grafted onto the surface of HNTs, which was in agreement with other reports [52]. The reason was that the strong hydrogen bond between the intersurface Al-OH inside the interlayers could block other molecules from inserting into the layers. Finally, the modified agents were grafted onto the silanol groups on the surface or the edge of the HNTs.

**Figure 3:**
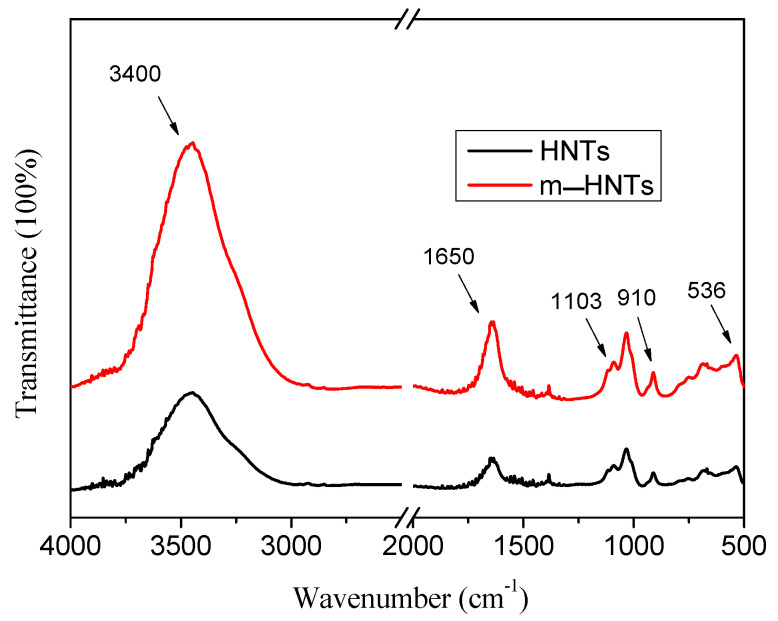
FTIR spectra of HNTs and m-HNTs.

Figure 3 shows the FTIR spectra of HNTs and m-HNTs. According to Figure 3, the peak at 1103 cm^−1^ corresponded to a Si-O stretching band and the peak at 910 cm^−1^ was attributed to an OH vibration band. The peak at 536 cm^−1^ was ascribed to an Al-O-Si band [53]. It could be seen that the intensity of two peaks at 3400 cm^−1^ and 1650 cm^−1^ increased. The broad peak of water OH stretch, centered at 3400 cm^−1^, was further increased in the spectra of m-HNTs samples, which is attributed to the overlap with the NH_2_ stretching vibration signal around 3400 cm^−1^. Meanwhile, the peak at 1650 cm^−1^ was ascribed to the OH deformation of water. The peak intensity was also increased owing to a superposition with a NH_2_ deformation vibration signal around 1620 cm^−1^.

**Figure 4:**
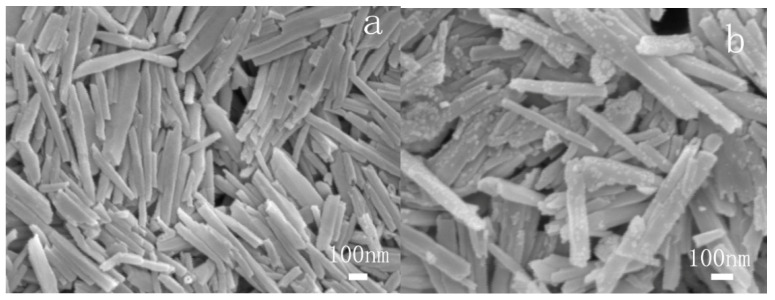
SEM images of HNTs (**a**) and m-HNTs (**b**).

Figure 4 shows the images of HNTs (a) and m-HNTs (b). It could be seen from (a) that the structure of the HNTs was tubular. The surface of the HNTs was smooth. According to (b), there are several obvious changes on the surfaces or the edges of m-HNTs compared to HNTs. The surface of m-HNTs became rough and some attachment was around the m-HNTs. The SEM images, accompanied with the FTIR and XRD results, confirmed that ODA molecules modified the HNTs.

### 3.3. Characterization of PI-HNTs Film

**Figure 5:**
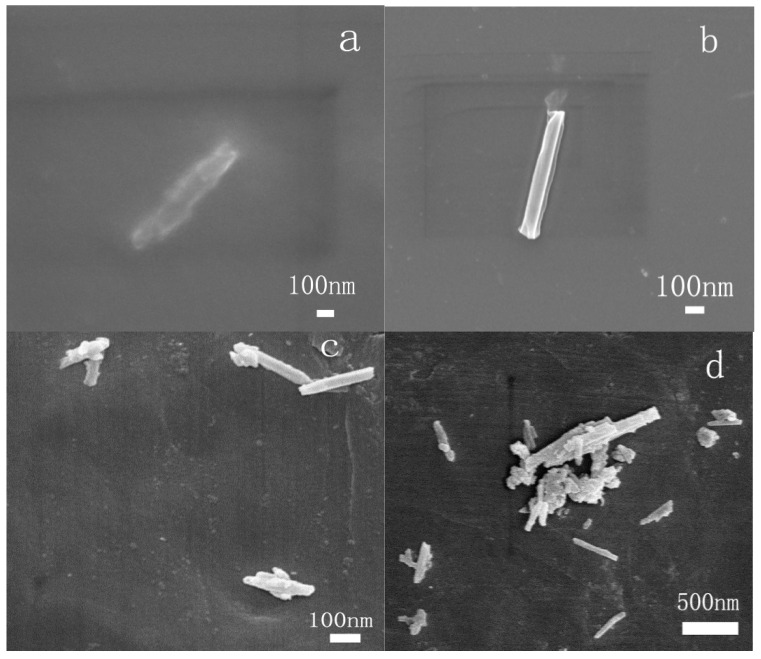
SEM of PI-HNTs-1% (**a**), PI-PHNTs-1% (**b**), PI-HNTs-3% (**c**), and PI-HNTs-5% (**d**).

Figure 5 shows the SEM images of PI-HNTs-1% (a), PI-PHNTs-1% (b), PI-HNTs-3% (c), and PI-HNTs-5% (d). According to the figure, the surface of m-HNTs in the PI-HNTs-1% was much more obscure and rougher than that of HNTs in the PI-PHNTs-1%. This is attributed to the polymer matrix covered on the surface. In addition, interfaces between PI and m-HNTs within PI-HNTs-1% were obviously different from that between PI and HNTs within PI-PHNTs-1%. The interface between PI and HNTs within PI-PHNTs-1% was clear. The phenomenon displayed that m-HNTs had better compatibility with bio-based polyimide film. This result confirmed that the modification of HNTs was beneficial for the compatibility between HNTs and bio-based polyimide. The reason was that the amino of the m-HNTs could be involved in the formation of a hydrogen bond and have a chemical reaction with bio-based polyimide film. According to Figure 5c, PI-HNTs-3%, and Figure 5d, PI-HNTs-5%, more HNTs aggregated to big particles with the increase in HNTs.

Table 1 shows the molecular weights and molecular weight distribution of PI, PI-PHNTs-1%, and PI-HNTs-1%. According to the table, the average molecular weight (Mw) and the number average molecular weight (Mn) of PI-PHNTs-1% and PI-HNTs-1% were higher than that of PI. This is because the HNTs and m-HNTs could easily adsorb small monomer molecules and increase the local concentration of the monomer. The increased concentration benefited the polymerization and could enlarge the Mw and Mn. It could also be found that the Mw and Mn of PI-HNTs-1% were higher than those of PI-PHNTs-1%. The reason is as follows: firstly, the hydrogen bond between the amino groups of the m-HNTs and those of bio-based polyimide film could easily be formed, which could further increase the adsorption of small molecules; secondly, compared to HNTs, m-HNTs had better compatibility with bio-based polyimide film and there were fewer large aggregates, so the hindering effect on the molecular movement was weak. It is easier for a small monomer or a short polymer chain to move across each other and react, thus prolonging the polymer chain. In addition, Mw/Mn of PI-PHNTs-1% and PI-HNTs-1% were less than that of PI, showing that the HNTs and m-HNTs could influence the molecular weight distribution.

**Figure 6:**
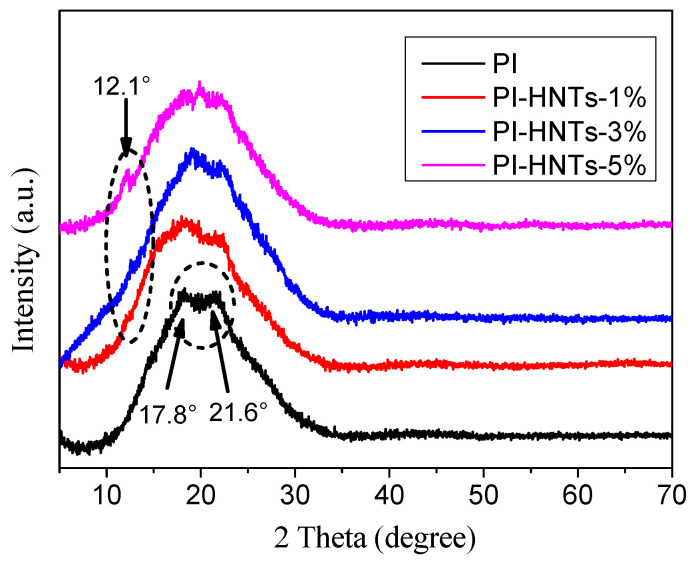
XRD patterns of PI, PI-HNTs-1%, PI-HNTs-3% and PI-HNTs-5%.

Figure 6 displays XRD patterns of PI, PI-HNTs-1%, PI-HNTs-3%, and PI-HNTs-5%. According to the figure, the typical diffraction peak at 2*θ* = 12.1° of HNTs was found in the curves of PI-HNTs-1%, PI-HNTs-3%, and PI-HNTs-5%. The peak intensity increased with the increase in the halloysite. Two typically wide diffraction peaks at 2*θ* = 17.8° and 21.6° of PI occurred on the curves of PI. At the addition of m-HNTs, the position of these two diffraction peaks changed. Meanwhile, these two wide diffraction peaks of PI gradually changed to a wide diffraction peak. The result showed that the m-HNTs could influence the crystal structure of the polyimide.

**Figure 7:**
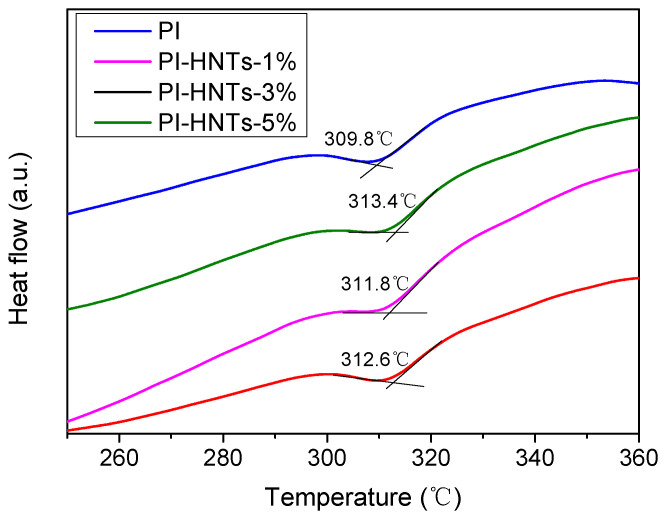
DSC of PI, PI-HNTs-1%, PI-HNTs-3%, and PI-HNTs-5%.

Figure 7 shows DSC of PI, PI-HNTs-1%, PI-HNTs-3%, and PI-HNTs-5%. As seen from the figure, Tg of PI-HNTs-1%, PI-HNTs-3%, and PI-HNTs-5% was 313.4 °C, 311.8 °C and 312.6 °C, which was larger than 309.8 °C of PI. The result displayed that the m-HNTs could influence the Tg of the bio-based polyimide film. This is because m-HNTs could restrict the movement of the bio-based polyimide chain. It could be noted that PI-HNTs-1% had the highest Tg. When the content of m-HNTs exceeded 1%, Tg of PI-HNTs-3% and PI-HNTs-5% decreased. The reason was that more m-HNTs could gather to aggregates, which could not be dispersed well.

**Figure 8:**
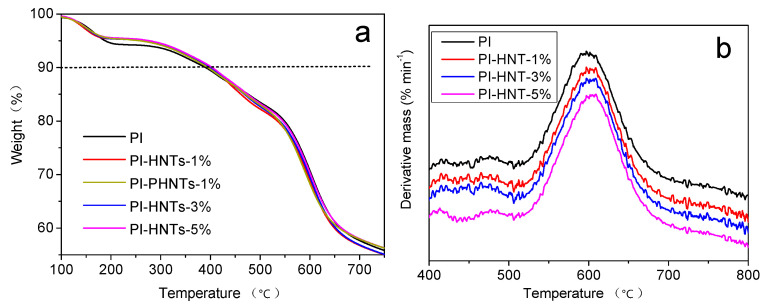
TGA (**a**) and DTG (**b**) curves of PI, PI-HNTs-1%, PI-HNTs-3%, and PI-HNTs-5%.

Figure 8 shows the TGA (a) and DTG (b) curves of PI, PI-HNTs-1%, PI-HNTs-3%, and PI-HNTs-5%. As shown in Figure 8a and Table 2, the temperatures of PI-HNTs-1%, PI-HNTs-3%, and PI-HNTs-5% were 397.1 °C, 399.0 °C, and 403.3 °C, which were higher than 390.1 °C of PI. According to Table 3, the peak temperatures on the DTG curves of the samples were 601.3 °C, 603.5 °C, and 608.6 °C, which were also larger than 596.9 °C of PI. The peak temperature was ascribed to the largest decomposition rate of the polymer. Therefore, the result shows that the thermal stability of PI-HNTs increased compared to PI. The reason is as follows: Firstly, when the temperature was high enough, small and volatile molecules could be released from PI-HNTs films. m-HNTs could absorb these molecules and prevent their escape, thus inhibiting the degradation of the polymer; Secondly, m-HNTs could absorb the heat and restrain the heat transfer; Thirdly, the good compatibility and physical or chemical interaction between m-HNTs and the polyimide film could further prevent the heat transfer and the diffusion of volatile molecules. In addition, it can be seen that the pristine HNTs could also improve the thermal stability of PI. This is because: firstly, HNTs could slow down the escape of volatile products in the degradation process due to barrier; secondly, volatile products may be entrapped into the lumen of Hal, causing an effective delay of mass transport and, consequently, increased thermal stability entrapment effects. Meanwhile, the temperature 10 wt% loss of PI-PHNTs-1% was lower than that of PI-HNTs-1%, showing that m-HNTs could improve the thermal stability of the PI more efficiently than pristine HNTs. The improved thermal stability is attributed to the barrier effect of the finely dispersed particles, which hindered the diffusion of small molecules generated during the thermal decomposition.

**Figure 9:**
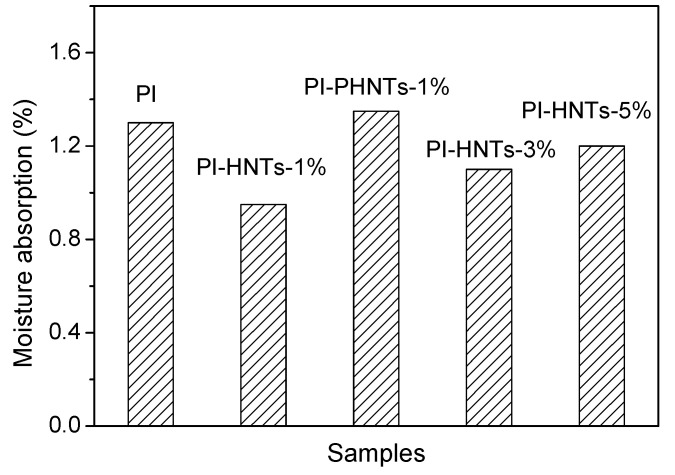
Moisture absorption of PI, PI-HNTs-1%, PI-PHNTs-1%, PI-HNTs-3%, and PI-HNTs-5%.

Figure 9 shows the moisture absorption of PI, PI-HNTs-1%, PI-PHNTs-1%, PI-HNTs-3%, and PI-HNTs-5%. The moisture of the bio-based polyamide film PI was 1.3%. After the addition of m-HNTs, the moisture absorption of PI-HNTs-1%, PI-HNTs-3%, and PI-HNTs-5% decreased. When the content of m-HNTs was 1%, bio-based polyimide/HNTs film had the least moisture absorption, showing the PI-HNTs-1% had the best water resistance. In order to compare the effort on the water resistance of bio-based polyimide film between pristine HNTs and modified HNTs, pristine HNTs were also added to the bio-based polyimide film to obtain PI-PHNTs-1%. Evidently, it could be seen that the moisture absorption of PI-PHNT-1% was higher than that of PI. It is reported that OH groups on the halloysite were hydrophilic and the interface defect could easily occur between the polyimide film and halloysite, resulting in the water absorption of PI-PHNTs-1% being higher than pure bio-based PI film. When the HNTs were modified, the introduction of ODA could improve the interface between the bio-based polyimide and HNTs. The interface defect could be reduced effectively. Therefore, the water resistance was improved. It is noted that water absorption of PI-HNTs-3% and PI-HNTs-5% increased compared to PI-HNTs-1%. This is because more aggregates occurred and made more defects.

Table 4 displays the results of tensile strength, strain at break, and Young’s modulus for the samples. It could be seen that the mechanical property of PI-HNTs-1%, PI-HNTs-3%, and PI-HNTs-5% was improved compared to pure bio-based polyimide film. It confirmed that m-HNTs could effectively enhance the mechanical property of the bio-based polyimide. According to the table, the tensile strength of bio-based polyimide film PI was 79.02 MPa. After the addition of m-HNTs, the tensile strength of PI-HNTs-1%, PI-HNTs-3%, and PI-HNTs-5% was increased to 108.85 MPa, 97.71 MPa, and 91.30 MPa, respectively. The tensile strength of PI-HNTs-1% was the highest, which increased by 37.7% compared to that of pure PI. This is attributed to good compatibility and interfacial interaction between m-HNTs and polymer matrix, which could reduce the stress concentration and thus afford much more stress distribution. Young’s modulus of PI was 1308 MPa. In contrast, Young’s modulus of PI-HNTs-1%, PI-HNTs-3%, and PI-HNTs-5% was 2290 MPa, 1380 MPa, and 1784 MPa, which increased by 75.1%, 5.5%, and 36.4%. This result showed that the PI-HNTs had stronger stiffness and the ability to resist the deformation. The strain at break of PI-HNT-1%, PI-HNTs-3%, and PI-HNTs-5% was 9.039%, 12.460%, and 8.829%, which increased by 22.0%, 68.2%, and 19.1% compared to 7.410% of pure PI. It is reasonable that the interfacial surface between m-HNTs and bio-based polyimide film was strong to improve the plasticity of the polyimide film.

## 4. Conclusions

The bio-based polyimide/HNTs nanocomposite based on 2,5-furandicarboxylic acid was successfully prepared. The HNTs were modified by TEOS and ODA. These two agents were grafted to the surface or the edge of the HNTs rather than intercalated into the interlayers. The surface of modified HNTs became rough compared to the pristine HNTs. The modified HNTs improved the water resistance and thermal stability of bio-based polyimide film. The tensile strength and Young’s modulus of PI-HNTs-1% were up to 108.85 MPa and 2290 MPa, increasing by 37.7% and 75.1% compared to those of pure PI. This work used the bio-based monomer to replace oil-based monomers successfully and supplied a method to prepare bio-polymer/HNTs nanocomposites.

## Figures and Tables

**Table 1:** The molecular weights and molecular weight distribution of PI, PI-PHNTs-1%, and PI-HNTs-1%.

Samples	Mw	Mn	Mw/Mn
PI	113,106	79,614	1.42
PI-PHNTs-1%	127,961	92,505	1.38
PI-HNTs-1%	135,636	98,947	1.37

**Table 2:** The decomposition temperatures of the samples at 10 wt% loss.

Samples	Temperature (°C) 10 wt% Loss
PI	390.1
PI-HNTs-1%	397.1
PI-PHNTs-1%	394.9
PI-HNTs-3%	399.0
PI-HNTs-5%	403.3

**Table 3:** The peak temperatures of the samples of PI, PI-HNTs-1%, PI-HNTs-3%, and PI-HNTs-5%.

Samples	Peak Temperature (°C)
PI	596.9
PI-HNTs-1%	601.3
PI-HNTs-3%	603.5
PI-HNTs-5%	608.6

**Table 4:** Values of tensile strength, strain at break, and Young’s modulus for all samples.

Sample	Tensile Strength (MPa)	Strain at Break (%)	Young’s Modulus (MPa)
PI	79.02	7.410	1308
PI-HNTs-1%	108.85	9.039	2290
PI-HNTs-3%	97.71	12.460	1380
PI-HNTs-5%	91.30	8.829	1784

## Data Availability

We declare that the data supporting the findings of this study are available within the article.
